# Prophylactic clipping prevents delayed bleeding after endoscopic mucosal resection of large non-ampullary duodenal lateral spreading lesions

**DOI:** 10.1055/a-2716-9623

**Published:** 2025-11-06

**Authors:** Gijs Kemper, Christian Gerges, Anton Jonkers, Torsten Beyna, Peter Siersema

**Affiliations:** 1 6034 Gastroenterology & Hepatology, Radboudumc, Nijmegen, Netherlands; 2 Department of Gastroenterology, Evangelisches Krankenhaus Düsseldorf, Düsseldorf, Germany; 3 6993 Gastroenterology & Hepatology, Erasmus Medical Center, Rotterdam, Netherlands

**Keywords:** Endoscopy Upper GI Tract, Endoscopic resection (ESD, EMRc, ...), Endoscopy Small Bowel, Neoplasia, Quality and logistical aspects, Performance and complications

## Abstract

**Background and study aims:**

Non-ampullary duodenal polyps account for a group of rare tumors of the gastrointestinal tract. Although small lesions are relatively easy to remove, resection of larger lesions requires more advanced techniques such as endoscopic mucosal resection (EMR). Although this technique is considered safe, the most prevalent complication is delayed bleeding (DB) with considerable incidence rates of up to 26%. In this study, we aimed to assess whether prophylactic clipping (PC) reduces DB rates after EMR of large duodenal non-ampullary lateral spreading lesions.

**Patients and methods:**

We retrospectively collected data from consecutive duodenal EMRs of non-ampullary lateral spreading lesions ≥ 15 mm performed between 2019 and 2022 at two medical centers in the Netherlands and Germany.

**Results:**

A total of 186 polyps with a mean size of 25 mm were included in this study. Most were tubular adenomas (55%) and contained low-grade dysplasia (84%). PC of the resection site was performed in 84 patients (45%). The overall DB rate was 13% (24/186). DB occurred in three of 84 cases with PC versus 21 of 102 cases without PC (4% versus 21%, <i>P</i> < 0.01). With an odds ratio of 0.22, multivariable analysis indicated that PC significantly reduced DB (95% confidence interval 0.06–0.85; <i>P</i> = 0.03).

**Conclusions:**

PC of the resection site significantly reduced DB after EMR of large non-ampullary duodenal lateral spreading lesions.

## Introduction

Non-ampullary duodenal adenomas are uncommon lesions that can be encountered sporadically or as part of a genetic syndrome 1 . Removal of these polyps is essential because 30% to 85% undergo malignant transformation 2 . Although duodenal lesions have traditionally been managed surgically, endoscopic resection has proven to be an effective and minimally invasive alternative 3 . While smaller polyps can be easily removed with cold snare polypectomy, the first-line endoscopic resection technique for larger non-ampullary lateral spreading lesions (LSLs) is endoscopic mucosal resection (EMR) 4 . Although EMR is regarded as a relatively safe procedure, it is not without risk. Adverse events (AEs) including intraprocedure bleeding (IPB), delayed bleeding (DB), and perforation are common and result in prolonged hospitalization 5 . DB is the most prevalent complication with overall incidence rates ranging from 5% to 13% 6 7 8 9 . These rates can even rise to 26% after resection of lesions > 30 mm 10 . To reduce DB, several prophylactic techniques have been advocated, including multiple topical hemostatic agents 11 , coagulation or clipping of visible vessels 7 , and prophylactic clipping (PC) of the resection site 12 . Robust studies reporting the effect of PC on DB following EMR of large duodenal polyps are limited. Our aim was to assess the effect of PC on DB rates after EMR of large (≥ 15 mm) non-ampullary duodenal LSLs.

## Patients and methods

### Study design

This was an international retrospective cohort study performed at the Radboud University Medical Center in Nijmegen, the Netherlands and the Evangelisches Krankenhaus in Düsseldorf, Germany. Due to the retrospective design of this study using anonymized patient data, ethical approval was waived (Regional ethical board CMO Arnhem-Nijmegen reference number 2021–13082).

### Patient selection

We collected data from all consecutive patients who underwent EMR for non-ampullary duodenal LSLs ≥ 15 mm between January 2019 and December 2022 with a 30-day follow-up. Patients with recurrent/residual lesions or lesions treated with endoscopic submucosal dissection (ESD) or EMR/ESD hybrid were excluded. Eligible patients were identified using local electronic health record search engines.

### Endoscopic procedure

**Fig. 1:**
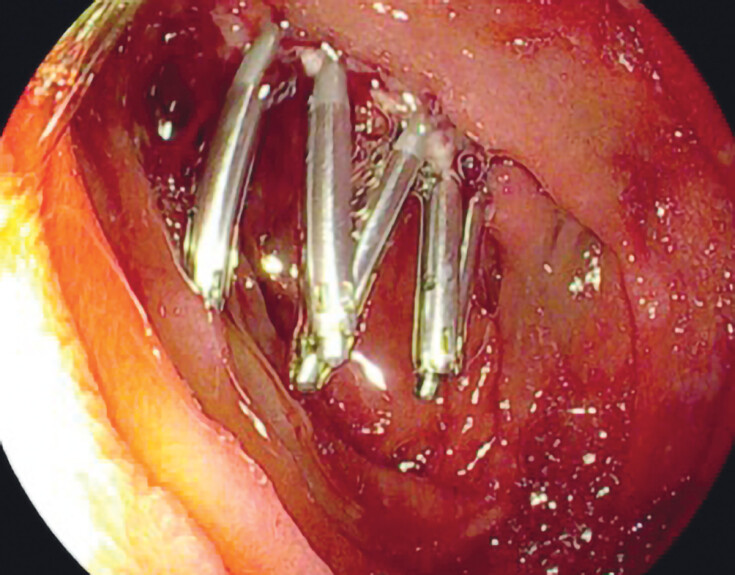
Duodenal post EMR-site closed with prophylactic clipping (PC).

All procedures were performed in accordance with local protocol at the endoscopy units of the respective hospitals. Technical aspects of the procedure including but not limited to type of scope used (without or without cap), piecemeal vs en bloc resection, lifting solution with or without epinephrine, and use of any prophylactic measures were at the discretion of the operator. PC was defined as any attempt to close the resection site with endoscopic clips ( Fig. 1 ). All patients were observed overnight before discharge and second-look endoscopies after EMR were performed according to endoscopist recommendations but not routinely. Anticoagulation drugs were paused and continued in accordance with local protocol.

### Outcome

The outcome of interest was DB defined as any suspicion of a bleeding event (hematemesis, melena, and hematochezia), or drop in hemoglobin levels of more than 1.5 mmol/L that required medical consultation, admission or prolonged admission in the absence of a more likely alternative diagnosis within 30 days after the procedure.

### Statistical analysis

All data were collected locally on standardized electronic case record forms (eCRFs) and analyzed using IBM SPSS statistics version 27. Data consisted of no more than one polyp per patient. Baseline characteristics were compared between groups (PC vs no PC). These tests were exploratory and used only to help identifying potential confounders. Categorical variables were described as frequencies and percentages. Continuous variables were depicted as means ± standard deviation. Comparison of categorical variables was performed using a χ2 or Fisher’s exact test when sample sizes were small. For comparing normally distributed continuous variables, a Student’s *t* test was used. Normality was assessed using visual inspection of Quantile-Quantile plots. Univariable analyses were conducted to identify potential risk factors for DB. In addition to PC, we selected patient and procedure related parameters that were likely to have impacted DB, including lesion size, piecemeal resection, IPB, anticoagulant use, and topical hemostatic agents. We used multivariable logistic regression to determine whether the association between PC and DB was affected by the imbalance of potential risk factors. Statistical significance was set at a threshold of 0.05 for all analyses.

## Results

### Patient and procedure characteristics

Over a 4-year period, 186 consecutive patients who met the inclusion criteria were treated with EMR. The majority were men (52%) with a mean age of 62 years. Most lesions were tubular (55%) or tubulovillous (37%) adenomas with low-grade dysplasia (84%) and were located in the second part of the duodenum (61%) with a mean size of 25 mm (± 14 mm). A total of 84 resection sites (45%) were treated with PC with a median of two clips per resection (range; 1–7). Polyps in the PC group were significantly smaller (mean size; 22 mm vs 27 mm) and were less often removed with piecemeal resection (60% vs 78%). Topical hemostatic agents were more frequently administered in the no PC group (43% versus 5%). Use of antithrombotic drugs, adjuvant thermal ablation of the resection margins, and snare tip soft coagulation of visible vessels was equally distributed between the two groups. Also, macroscopic complete resection and IPB rates were similar ( Table 1 ).

**Table:** **Table 1** Baseline patient and procedure characteristics.

	**PC (N = 84)**	**No PC (N = 102)**	***P* value **
Age, years, mean (SD)	62 (± 15)	62 (± 15)	0.89
Sex			0.81
Female	41 (49)	48 (47)	
Male	43 (51)	54 (53)	
Antiplatelet agents	19 (23)	24 (24)	0.92
Anticoagulation agents	10 (12)	9 (9)	0.49
Lesion size, mean, mm (SD)	22 (±10)	27 (±16)	< 0.01
Location			0.08
D1	11 (13)	27 (27)	
D2	55 (66)	58 (60)	
D3	18 (21)	17 (17)	
Piecemeal resection	50 (60)	79 (78)	0.02
Macroscopic complete resection	79 (94)	89 (87)	0.12
Intraprocedural bleeding	22 (26)	23 (23)	0.56
Adjuvant thermal ablation margins	9 (12)	14 (14)	0.72
STSC visible vessels	2 (2)	3 (3)	0.81
Topical hemostatic agents	4 (5)	44 (43)	< 0.01
Histopathology			0.66
None	2 (2)	0 (0)	
Tubular	47 (57)	55 (54)	
Tubulovillous	29 (35)	39 (38)	
Villous	1 (1)	2 (2)	
Sessile serrated	2 (2)	1 (1)	
Cancer	1 (1)	0 (0)	
Hyperplastic	2 (2)	5 (5)	
Dysplasia			0.74
None	5 (6)	6 (6)	
Low-grade dysplasia	70 (83)	87 (85)	
High-grade dysplasia	8 (10)	9 (9)	
Cancer	1 (1)	0 (0)	
Values are frequencies (%) unless otherwise indicated. PC, prophylactic clipping; SD, standard deviation; STSC, snare tip soft coagulation.			

### Adverse events

AEs occurred in 27 of 186 resections (14%) with 24 of 186 being DB events (13%), two perforations, and one post-polypectomy syndrome. The majority of the DBs (75%) were diagnosed within 2 days after the procedure. Twenty of 24 patients (83%) required additional endoscopy with a median prolonged hospital admittance of 3 days. Thirteen patients were treated with subsequent clipping with or without use of topical hemostatic agents, whereas seven patients were treated with topical hemostatic agents only. Three patients required blood transfusion. DB was present in three of 84 patients with PC vs 21 of 102 patients without PC (4% versus 21%, *P* < 0.01). There were no differences in perforation rates, PPS rates, or intensive care unit admission rates between the two groups ( Table 2 ).

**Table:** **Table 2** Adverse events.

	**PC (N = 84)**	**No PC (N = 102)**	***P* value **
DB	3 (4)	21 (21)	< 0.01
Perforation	2 (2)	0 (0)	0.20
PPS	1 (1)	0 (0)	0.46
ICU admission	3 (4)	5 (5)	0.73
Values are frequencies (%) unless otherwise indicated. DB, delayed bleeding; ICU, intensive care unit; PC, prophylactic clipping; PPS, post-polypectomy syndrome.			

### Risk factors

On univariable analysis, use of PC appeared to reduce DB (relative risk 0.17, 95% confidence interval [CI] 0.05–0.56; *P* < 0.01), whereas presence of IPB (relative risk [RR]2.24, 95% CI 1.10–4.69; *P* =0.03), lesion size ≥ 30 mm (RR 2.82, 95% CI 1.31–6.06; *P* < 0.01) and application of topical hemostatic agents (RR 3.40, 95% CI 1.63–7.10; *P* < 0.01) was associated with higher DB rates ( Table 3 ). After controlling for presence of IPB, lesions ≥ 30 mm and topical hemostatic agents in the multivariable analysis, PC (odds ratio 0.22, 95% CI 0.06–0.85; *P* = 0.03) remained a statistically significant factor for preventing DB ( Table 4 ).

**Table:** **Table 3** Univariable analysis identifying potential delayed bleeding risk factors.

	**RR**	**95% CI**	***P* value **
Piecemeal resection	2.21	0.79–6.17	0.11
Lesion size ≥ 30 mm	2.82	1.31–6.06	< 0.01
IPB	2.24	1.10–4.69	0.03
PC	0.17	0.05–0.56	< 0.01
Topical hemostatic agents	3.40	1.63–7.10	< 0.01
Antiplatelet agents	1.65	0.76–3.59	0.21
Anticoagulation agents	0.80	0.20–3.14	0.74
CI, confidence interval; IPB, intraprocedural bleeding; PC, prophylactic clipping; RR, relative risk.			

**Table:** **Table 4** Multivariable logistic regression model estimating the association between PC and delayed bleeding, adjusted for covariates.

	OR	95% CI	P value
PC	0.22	0.06–0.85	0.03
Covariates:			
Lesion size ≥ 30 mm			
IPB			
Topical hemostatic agents			
CI, confidence interval; IPB, intraprocedural bleeding; OR, odds ratio; PC, prophylactic clipping.			

## Discussion

This study demonstrates that closing the resection site with PC reduced DB rates after EMR of large non-ampullary duodenal LSLs. DB is the most common EMR-related complication throughout the gastrointestinal tract 13 14 15 16 . Evidence about the effect of PC in reducing DB is ambiguous. Two studies have evaluated the effect of PC after gastric endoscopic resection with contrasting results 17 18 . In the colorectum, PC is considered to only reduce DB risk after EMR in a selective group of patients with large (≥ 20 mm) proximal lesions 19 20 . PC in the duodenum is potentially more beneficial due to higher DB rates. In our study focusing on LSLs ≥ 15 mm, we found a substantial DB rate of 13%. This is in line with previously reported studies in which larger lesion size was identified as a DB risk factor 6 9 . Rates are thought to be higher because of the thin wall and the extensive vascular supply in the submucosal layer 10 . In addition, mucosal exposure to bile acids and pancreatic fluids might impair wound healing. In this study, we observed a DB rate reduction of 21% to 4% ( *P* < 0.01) when performing PC. In contrasting, use of topical hemostatic agents was associated with increased DB, acting most likely as a confounder because these agents were almost exclusively used in the no PC group. Lesion size ≥ 30 mm and IPB were also found to increase DB risk but were not identified as independent risk factors in multivariable analysis.

The number of previous studies available assessing the effect of PC after solely EMR in the duodenum is limited. In 2008, Lépilliez and colleagues analyzed the effect of PC and/or argon plasma coagulation of the entire resection surface and found a DB rate of 0% vs 22% when no prophylactic measures were performed. This study was limited by its small sample size of 37 lesions 21 . More recently, a handful of other studies have published similar results but also included lesions resected with endoscopic resection techniques other than EMR 22 23 24 . In contrast, a study including 167 non-ampullary duodenal adenomas ≥ 10 mm reported significantly fewer DB events in the absence of PC (29.3% vs 15.3%, *P* = 0.003). However, PC was not associated with DB in multivariate analysis. The authors state that the non-beneficial effect is likely the result of including a relatively large portion (45%) of lesions > 30 mm, because failure to completely close the resection site is associated with increased lesion size 5 . Other preventative measures including topical hemostatic powders or gels have been advocated 11 . Unfortunately, and in accordance with our findings, the effectiveness of these agents in reducing DB rates has not been reported so far.

Strengths of our study were the high number of large lesions, consecutively resected in one academic and one non-academic hospital. This study, however, is mainly limited by its retrospective non-randomized design, which may have resulted in allocation bias regarding PC. In addition, due to the unavailability of data on the degree of clip closure, we were unable to compare DB rates after complete clip closure versus partial closure.

## Conclusions

In conclusion, our findings suggest that PC of the resection site reduces DB after EMR of large, non-ampullary, duodenal lateral-spreading lesions. Prospective studies are required to validate these results and to identify high-risk patients for whom PC could be cost-effective.
